# Pathological Findings and Oxidative Stress Status Associated with Hydatidosis in Dromedary Camels

**DOI:** 10.3390/vetsci10020074

**Published:** 2023-01-19

**Authors:** Salma A. Shoulah, Mohamed M. S. Gaballa, Marawan A. Marawan, Sayed A. Saqr, Abdelhamed Abdelhady, Hayat Ali Alzahrani, Majed H. Wakid, Omar A. Al-Jabr, Abdelfattah Selim

**Affiliations:** 1 Department of Animal Medicine (Infectious Diseases), Faculty of Veterinary Medicine, Benha University, Toukh 13736, Egypt; 2 Department of Pathology, Faculty of Veterinary Medicine, Benha University, Toukh 13736, Egypt; 3 General Supervisor of Al-Basateen Abattoir of Cairo Veterinary Authority, Cairo 4252011, Egypt; 4 Department of Parasitology and Animal Diseases, National Research Centre, Dokki, Giza 12622, Egypt; 5 Department of Medical Laboratory Technology, Faculty of Applied Medical Sciences, Northern Border University, Arar 91431, Saudi Arabia; 6 Department of Medical Laboratory Technology, Faculty of Applied Medical Sciences, King Abdulaziz University, Jeddah 21589, Saudi Arabia; 7 Special Infectious Agents Unit, King Fahd Medical Research Center, King Abdulaziz University, Jeddah 21589, Saudi Arabia; 8 Department of Microbiology, College of Veterinary Medicine, King Faisal University, P.O. Box 400, Al-Asha 31982, Saudi Arabia

**Keywords:** antioxidant activity, camels, epidemiology, Egypt, hydatid cyst, histopathology

## Abstract

**Simple Summary:**

Cystic echinococcosis is a zoonotic helminthic disease that causes severe economic losses. The prevalence of hydatidosis was 21.7% in examined camels. Camels’ liver infections were rare, whereas their lung infections were more common. By comparing to non-infected camels, the level of malondialdehyde (MAD) was significantly increased with hydatid cysts infection, while the levels of reduced glutathione (GSH), superoxide dismutase (SOD), and (catalase) CAT were significantly decreased. The histopathological sections of camel cyst revealed layered membranes surrounded by a zone of cellular infiltration and an outermost fibrous tissue reaction. In addition, there was evidence of atelectasis, emphysema, hemorrhage, congestion, and fibrosis in the surrounding tissues.

**Abstract:**

(1) Background: Cystic echinococcosis is a zoonotic helminth disease that causes severe economic losses. The study aimed to assess the prevalence and viability of cystic echinococcosis in examined camels. In addition, assessing the histological, morphological, oxidative, and antioxidant state related to the cystic echinococcosis infection; (2) Methods: The study was performed on 152 slaughtered dromedary camels between March and September 2022 at El-Basatin abattoir in Cairo Governorate, Egypt; (3) Results: The results revealed that the prevalence of hydatidosis was 21.7% in slaughtered camel and the highest infection rate observed in lungs was 87.87%, while it was 9% in livers. Camels’ liver infections were rare, whereas their lung infections were more common. By comparing to non-infected camels, the level of MAD was significantly increased with hydatid cysts infection, while the level of GSH, SOD and CAT was significantly decreased. Histopathological section of camel cyst revealed layered membranes surrounded by a zone of cellular infiltration and an outermost fibrous tissue reaction. In addition, there was evidence of atelectasis, emphysema, hemorrhage, congestion, and fibrosis in the surrounding tissues. Nonetheless, the degeneration and necrosis of hepatocytes and other pathological alterations in liver cyst sections were remarkably comparable to those seen in the lungs. Furthermore, calcification was detected.

## 1. Introduction

Camels (*Camelus dromedarius*) are regarded as the “ship of the desert” due to their exceptional adaptation to hot and arid environments. In addition, camels are valuable livestock animals in many African countries, especially those in dry and semi-dry regions [1,2,3,4]. Egyptian camels produced 20.8 tons of milk, 2.3 tons of meat, 0.62 tons of hide, and 0.09 tons of fiber in 2017 [5]. Camel milk is better for people with allergies, while camel meat and milk are lower in cholesterol and fat than other livestock products [6].

Food security is a key concern on a global scale as it is linked to the objective of sustainable development. In order to respond to the growing population and to increase awareness of the consequences of climate change, the FAO and WHO prioritized food security in 2018 [7].

Moreover, Egypt is suited to a variety of cattle diseases due to its diverse environment and various agro-climatic zones. It has been stated that a wide range of different internal and exterior parasitic infections are the main problems influencing the health, productivity, and performance of domestic animals [8,9,10,11,12]. The parasite infestation can have negative economic and public health effects, with the most obvious economic loss resulting from the condemnation of viscera and sometimes carcasses, as well as lowered meat, wool, and milk production [13,14]. Moreover, it has been found that several parasites are responsible for food-borne diseases, which can cause mild discomfort or debilitating and even fatal diseases. Among the most important parasitic diseases is hydatidosis, which caused by *Echinococcus granulosus* sensu lato (s.l.) and is indirectly transmitted to human [15]. In general, the majority of published reports show that Egypt has a low endemicity of human cystic echinococcosis (CE). Human CE occurs in the majority of Egypt; however, the data that are now available are primarily from northern Egypt, specifically Cairo and Giza [16,17]. The illness is probably underdiagnosed or underreported in southern Egypt. In the Egyptian population, living in rural areas, working as a farmer, and getting older were all important risk factors for CE [18].

Even though the disease in animals is usually asymptomatic and only identifiable from hydatid cysts during post-mortem inspection in the abattoir, it causes significant economic losses [19]. Hydatid cyst is composed of three layers filled with a clear yellow fluid containing many protoscoleces [20]. In hydatid fluid, proteins (albumin and globulin), minerals (sodium, potassium, and zinc), and free amino acids have been found [21]. In the case of cyst-infested organ removal, hydatid fluid may leak out and possibly contaminate the carcass with cyst contents [22].

Many diseases, as well as parasitic infections, are accompanied by the generation of reactive oxidative species as a result of oxidative stress [23,24,25]. In addition, the pathogenesis of the oxidative stress reaction seen in the host tissues is influenced by the free radicals produced due to parasite infections [26]. It is indirect, but reliable, to estimate free radical activity and oxidative stress reaction by measuring antioxidant enzyme activities and endogenous antioxidants in blood [25]. In addition to providing insight into the pathogenesis of the disease, such information will provide an indication of parasitized animals’ oxidative response.

In all inhabited continents, including sub-arctic, arctic, temperate, subtropical, and tropical zones, echinococcosis is one of the most geographically prevalent zoonotic illnesses. It is most common in underdeveloped and developing countries. In agricultural countries throughout Europe; northern, eastern, and southern Africa; southern and northern America; the Middle East; and Asia, the illness is endemic to hyperendemic [27,28].

Additionally, hydatidosis can affect a variety of animal species [29]. In Morocco, the prevalence of hydatidosis was 12.03% in camels, 10.58% in sheep, and 22.98% in cattle [30], while 13.9% of cattle and 24.8% of camels in Algeria were infected [31]. Moreover, in Libya, the prevalence of hydatidosis was 4.9% in sheep, 2.4% in goats, 2.7% in camels, and 15% in cattle [32], and it was ranged from 16.42% to 40.42% in sheep, 8.56% in cattle, 6% in dromedaries, 2.9% in goats, and 8.48% in donkeys in Tunisia [33].

In Egypt, hydatid cysts have been observed in a variety of hosts. For instance, El Kordy [34] found 31% of camels, 10% of cattle, and 1.5% of sheep at a Cairo slaughterhouse had the illness. In addition, he discovered calcified cysts in 36.4% of the studied animals. Abdou [35] observed 30% infection in camels, 10% in cattle, and 5% in sheep at the same slaughterhouse, with significantly lower levels at an Alexandria slaughterhouse [36]. Additionally, 3.4% of the camels at the Cairo abattoir had hydatid cyst infections, according to El-Refaie et al. [37]. Another study on the prevalence of hydatid disease in butchered camels discovered that 26.5% of them were infected at Assiut abattoirs between 1985 and 1989 [38]. Additionally, Dyab et al. [39] found 7.67% of slaughtered camels in the Assuit and Aswan governorates were infected with hydatid cyst but did not observe in cattle or buffaloes. Moreover, El-Dakhly et al. [40] discovered CE in 62 (10.82%) camels, 33 (0.77%) sheep, and 3 (0.24%) pigs at the El-Basatin slaughterhouse in Cairo.

This is due to the fact that the fluid found within the cysts helps us to understand how hydatid cysts form within the body. Understanding the oxidative stress reaction response will help with control measures and reduce the likelihood of disease transmission [26,40].

The present study aimed to determine the prevalence of hydatidosis in slaughtered camels at the El-Basatin abattoir, Cairo, as well as to investigate histopathological lesions and changes oxidatively associated with infestation by hydatid cysts.

## 2. Materials and Methods

### 2.1. Ethical Statement

All procedures, including the handling and collection of blood samples, were approved by the Benha University ethical committee for animal studies (Approval No: BUFVTM 13-03-22).

### 2.2. Animals and Sampling

The study was carried out at El-Basatin abattoir between March 2022 and September 2022 to ascertain the prevalence and histological alterations connected with hydatidosis-induced liver and lung diseases in camels. A total of 152 camels were slaughtered in accordance with government regulations and subjected to postmortem examination. The slaughtered camels were previously living and kept at Cairo and Giza governorates, Egypt by individual farmers. Several incisions were made in each liver and lung after palpation. Cysts and other abnormalities were inspected and identified with an in-depth visual examination. Immediately after the animals were slaughtered, blood samples were obtained. The collected blood samples were centrifuged at 3000 rpm/15 min, then divided into small aliquots and stored at −20 °C for further analysis. The corresponding livers and lungs of the slaughtered animals were visually examined, and tissue specimens were collected for histopathological examination. Data regarding age and sex of examined camels were collected.

### 2.3. Hydatid Cyst Categorization

According to previously described protocol by Gareh et al. [41], hydatid cysts were examined morphologically to determine their shape, size, viability, and condition. Hydatid cysts were classified into three categories based on their characters: fertile hydatid cysts, which contain protoscoleces and/or daughter cysts; sterile hydatid cysts, which are fluid filled but lack protoscoleces; and calcified hydatid cysts, which lack protoscoleces or fluid and have a tough thickened wall. An aspiration needle of 21 gauge was used to extract the hydatid fluid from each cyst. After collecting the fluid, it was centrifuged at 252× *g* for five minutes. In the next step, the last drops of sediment were transferred to slides, mounted with a glass cover slip, and examined under a microscope for protoscoleces, brood capsules, and taeniid hooks.

### 2.4. Biochemical Analysis of Cyst Fluid

The fluid from separate cysts was extracted with a sterilized syringe and centrifuged at 10,000× *g* at 4 °C for 30 min. The supernatant was gathered and utilized to estimate calcium (Ca), inorganic phosphorous, and sodium (Na) potassium (K) according to [42,43,44], respectively, utilizing suitable kits (Biodiagnostic, Cairo, Egypt).

### 2.5. Histopathological Examination

In lung and liver samples, paraffin-embedding techniques were used to demonstrate pathological changes [45]. The histopathological procedure involves fixing tissues in neutral formaldehyde buffer, dehydrating them in degraded alcohol concentrations, clearing them in xylol, embedding them in paraffin wax, and sectioning them into 4-micron-thick sections that are stained with hematoxylin–eosin (HE) for microscopic examination.

### 2.6. Oxidative State Evaluation

The measurement of malondialdehyde (MDA), glutathione peroxidase (GSH-PX), superoxide dismutase (SOD), and catalase (CAT) in serum samples were assessed using a commercial kit (Biodiagnostic, Cairo, Egypt), following manufacturer instructions.

### 2.7. Statistical Analysis

The data were analyzed using SPSS software version 24 (IBM, New York, NY, USA). Descriptive statistics, such as percentage, were calculated and the prevalence of hydatidosis in camels of different ages and sexes. The data obtained from hydatidosis-infected camels and control group were compared using independent t-test and differences were considered statistically significant if *p* < 0.05.

## 3. Result

### 3.1. Prevalence of Hydatidosis in Camels

Out of 152 examined camels, 33 (21.7%) were infected with hydatid cysts. Moreover, the prevalence of hydatidosis was non-significantly (*p* > 0.05) higher in females (25.71%) and camels less than 4 years old (22.5%) than other camels, Table 1.

### 3.2. Parasitological and Biochemical Analysis of Hydatid Cyst

**Figure 1:**
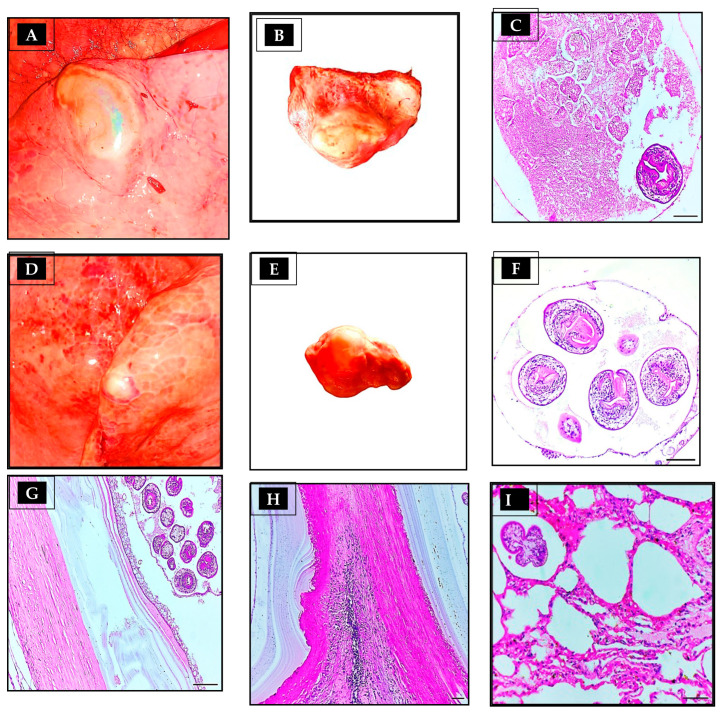
Morphological appearance of hydatid cyst in the lung of camels. (A) Camel lung with intact, (**A**,**B**) medium size, and (**D**,**E**) small size tumor-like fluid-filled hydatid cysts, enveloped by a fibrous capsule. (**C**,**F**) Cross-section of an hydatid cyst, stained with H&E displaying brood capsule and scolex holding multiple hooklets (thick arrow); (**G**) Lung section viewing fibrous tissue capsule infiltrated with inflammatory cells, parasitic membranes, and scolices. (**H**) Camel lung section of camel origin hydatid cyst showing the laminated membrane, the fibrous tissue capsule infiltrated with inflammatory cells. (**I**) Lung section of camel origin showing existence of parasitic sections in the lung tissue.

Usually, a hydatid cyst in the lung classified as *E. granulosus* sensu lato (s.l.), which looks like a cotton ball, implanted in the parenchyma (Figure 1A,D), filled with clear to slightly turbid fluid, soft and malleable to touch, and contained within the white germinal layer (Figure 1B,E); by contrast, when it was about to be removed, a liver hydatid cyst was firm and calcified. Unlike protruding cysts, these cysts were found deep within the liver tissue and were very small. The predilection site of hydatid cyst in camels seems to be the lung as 29 (87.87%) cysts were in lungs, 3 (9%) cysts were in livers and 1 cyst (3.03%) was in the liver and lung, Table 2.

All collected cysts were small or medium in size, no large cysts were found among the collected samples, where 17 (51.52%) cysts were small and 16 (48.48%) cysts were medium in size. Additionally, cyst fluid ranged from 4 to 14 mL. Moreover, 25 (75.76%) cysts had watery fluid consistency and 8 (24.24%) cysts had thick consistency. Watery fluids were clear, whereas thick fluids were turbid or had a yellowish color. Additionally, 19 (57.58%) cysts were fertile, and 14 (42.42%) cysts were sterile, Table 2.

The levels of sodium and potassium concentration in examined camels ranged between 34.8 to 68.1 mmol/L and 0.3 to 2.2 mmol/L, respectively. In addition, the levels of calcium and phosphorus concentration in infected camels ranged between 9 mmol/L to 84 mg/100 mL and 0.4 to 4.8 mg/100 mL, respectively, Table 2.

### 3.3. Antioxidant Activity

Regarding the changes in the antioxidant status of infected camels. There was a significantly (*p* < 0.0001) increased level of MAD in infected camels, while GSH-PX, SOD, and CAT were significantly (*p* < 0.0001) decreased in infected camels compared to the control group, Table 3.

### 3.4. Histopathological Findings

**Figure 2:**
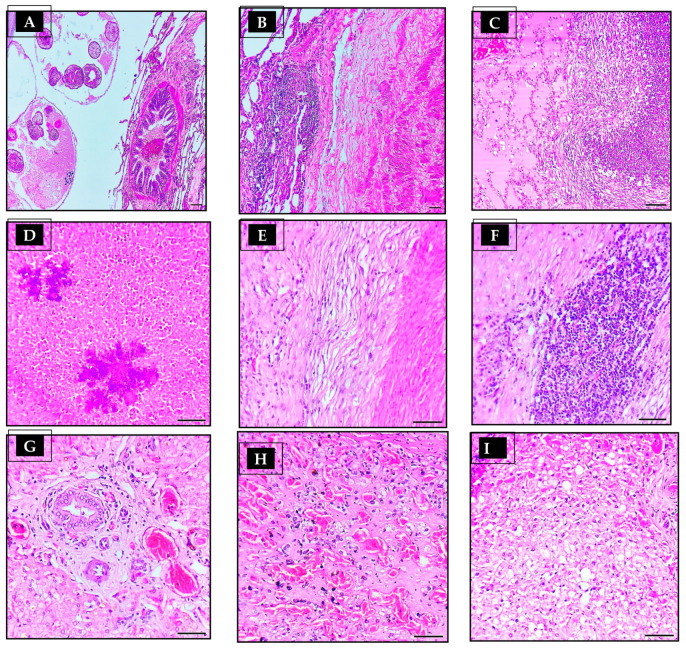
Histological characteristics seen with hydatid cyst from neighboring tissues of both lungs and livers. (**A**–**C**) Lung section showing (**A**) hyperplasia of the bronchiolar epithelium, and the presence of intra-bronchial inflammatory cells. (**B**) Fibrous tissue capsule, zone of infiltrated inflammatory cells, collapsed lung tissue, and compressed bronchiole. (**C**) Collection of inflammatory cells forming granuloma and collapsed lung tissue, as well as oedema in adjacent pulmonary parenchyma. (**D**–**I**) Liver section of camel origin hydatid cyst showing (**D**) area of necrosis intermixed with dystrophic calcification surrounded with (**E**) fibrous capsule and (**F**) heavy cellular reaction, including lymphocytes, eosinophils, and histiocytes. (**G**) Liver section showing fibrosis in the portal area with proliferation of small capillaries and (**H**) severe congestion of hepatic sinusoids, as well as (**I**) vacuolar and fatty degeneration of hepatocytes are seen in adjacent liver parenchyma.

In general, there were three layers associated with the hydatid cyst structure: an external fibrous layer (pericyst), a middle acellular eosinophilic laminated membrane layer (ectocyst), and an internal germinal layer (endocyst) (Figure 1C,F). When examined histopathologically, all lung sections showed fibrous tissue reactions (Figure 1G), cellular reactions (Figure 1H), and collapsed lung tissue around the cyst wall (Figure 2B). The liver sections, on the other hand, showed fibrous tissue reactions (Figure 2E), cellular infiltration (Figure 2F), hepatocyte necrosis, and calcification near the cyst walls (Figure 2D).

In hydatid-cyst-infected lung histology, there were varying numbers of parasitic sections (scolices) in lung tissue (Figure 1I, but none were found in liver tissue. During an inflammatory reaction to a hydatid cyst layer, epithelioid macrophages, lymphocytes, and eosinophils were infiltrated in the fibrous layer (Figure 2F). The extent of cellular infiltration reduced as they distanced themselves from hydatid cysts. The majority of inflammatory cells infiltrating the body are mononuclear cells, macrophages, lymphocytes, plasma cells, and neutrophils (Figure 2C). When cysts reach substantial sizes, lung tissue collapses, subsequent emphysema, and bronchial compression (Figure 2B). The proliferation of bronchial epithelium was evident on some slides (Figure 2A), whereas lung congestion and hemorrhage were evident on others. Among the remaining lung tissue, certain sections had chronic pneumonia, suppurative bronchitis, and peribronchial gland proliferation.

Upon microscopical examination of hepatic tissue, the cysts appeared to have been filled with caseated and calcified material. This material was surrounded by hemorrhage (Figure 2G,H), hepatocyte degeneration (Figure 2I), cytoplasmic swelling, a fibrous capsule, and mononuclear inflammatory cells.

## 4. Discussion

In Egypt, the routine monitoring of camel hydatidosis is a crucial requirement for providing updated information about the disease in animals along with people. Therefore, the present study aimed to evaluate histological, morphological, oxidative, and antioxidant state related to the hydatidosis infection.

The overall prevalence of hydatidosis in this study was 21.7%. To ascertain the prevalence of hydatidosis in camels in other countries, numerous studies have been carried out. In eastern Ethiopia, the prevalence rate was 18.8% [46]; in Saudi Arabia, it was 32.85% [47]; in Egypt, it was 7.67% [39]; and in China, it was 49.3% [48]. According to another study, 48% of the camels in Libya had *E. granulosus* [49]. Additionally, In Kuwaiti camels, the prevalence rate was 39.65% [50]. The reported prevalence rate variances are predicted matter for a number of reasons, such as variations in environmental and climatic conditions. This change could be explained by improved environmental factors that are favorable for parasite survival [51,52,53,54].

The prevalence of infection was non-significantly higher among older camels (>4 years) in comparison with young ones (<4 years). The current findings corroborated the observation of Ahmed et al. [55] that hydatid cyst infections become more prevalent as camels age, and Elham et al. [56] reported that the cysts of *E. granulosus* are more prevalent in Iranian camels older than 15 years in comparison with young ones. As previously observed by Mirzaei et al. [57], the prevalence of hydatid cyst increased with age, where it was 5.6% in animals aged 5–10 years, with a lower frequency in younger animals (2.02%).

Age variations in the livestock may account for the difference in exposure, since older animals may have been exposed to higher infective stages [58]. Additionally, this makes sense because cysts in younger animals are still in their formative stages and need time to mature [59].

Hydatid cyst infection was found to be greater in females (21.42%) than in males (18.29%), correlating with the previous studies of Mirzaei et al. [57] but contrary to findings obtained by Fathi et al. [60]. Contrary to present findings, Ahmadi [61] observed that the prevalence of hydatidosis was non-significantly higher in males (34.4%) than females (36.6%).

The metabolism, physiology, and immunogenicity of hydatid cysts are all significantly impacted by the presence of minerals and organic components [62]. These chemicals also control the activity of gamma glutamyl transpeptidase, a membrane enzyme essential for the export of amino acids and peptides from the cell [63]. Sharif et al. [64] found statistically significant differences in potassium and calcium levels between cysts in sheep, goats, cattle, and camels, but not changes in sodium levels, in Iran. It is well established that parasites cause lipid peroxidation and free radical production in host organs, tissues, and cells [65]. The surface of a parasite is attacked by free radicals released by activated phagocytes. The activation of macrophages occurs via cytokines released by activated lymphocytes. These free radicals operate as effectors of resistance but do so in a nonspecific manner [66]. Oxidative damage is caused by free radicals, such as hydroxyl, superoxide anion, and hydrogen peroxide. The production of cytosolic aldehyde and peroxide products is a sign that oxidative damage has occurred, which, in turn, causes a release of cell and organelle contents and a loss of important fatty acids. MDA is the byproduct of free radical reactions on lipids in cell membranes [23]. Antioxidant systems, such as SOD, CAT, and GSH, protect cells from the damaging effects of free radicals against lipid peroxidation generated by parasites [67]. The initial line of antioxidant defense in a cell is mostly mediated by the enzyme superoxide dismutase (SOD). The enzyme neutralizes the superoxide radical. CAT detoxifies the hydrogen peroxide generated from SOD activity. Hydrogen peroxide can also be broken down by CAT, which then produces water [68].

The present results demonstrated that MAD was significantly upregulated; meanwhile, SOD, CAT, and GSH were all down regulated in infected camels compared to non-infected camels. These findings come in accordance with those of Samadieh et al. [69] who found an elevated serum MDA concentration in infected sheep from Iran due to *Dicrocoelium dendriticum* infection. These infection causes substantial alterations in the oxidative status of sheep in the Khuzestan province, Iran, as indicated by an increase in MDA concentration [23]. Furthermore, it was consistent with Sarin et al. [70] who found that parasite infections raise free radicals, which, in turn, produce membrane lipid peroxidation and cellular damage in host cells.

Postmortem and slaughterhouse meat inspection necropsies are often regarded as the gold standard for diagnosing intermediate animals [71]. In the present study, the highest prevalence rate of hydatid cyst was observed in the lungs (87.87%), while the lowest rate was in the liver (9.0%), and in both organs it was around 3.03%, which was consistent with the results of previous studies [60]. A similar pattern of results was observed in camels in the Assuit and Aswan governorates, Egypt, as reported by Dyab et al. [39], as well as in camels from the Mansoura and Cairo governorates, Egypt, as documented by Haridy et al. [72] and Rahman et al. [73].

The higher infection rate in lungs, comparable to liver, could be explained by the oncosphere being more likely to remain in any organ it encounters first and the size of the *E. granulosus* oncosphere in relation to the venules and lymphatic lacteal of the villus in different animals. Lung cysts are more prevalent in ruminants because of the size of the lymphatic lacteal of the villus. However, liver cysts are more common in non-ruminants because their lymphatic systems are much smaller [74]. Contrary to the present findings, other researchers observed that hydatid cysts more common in livers (75%) than lungs (17%) in camels from the Assuit governorate, Egypt [75]. This might be because the liver is the first organ the big metacestode remains in after entering the bloodstream through the mucosa of the gut [76].

The findings of present study revealed that the majority of cysts were either small (1–5 cm) or medium (6–10 cm) in size, with 51.52% of cysts falling into the former category and 48.48% in the latter, and most of cysts (57.58%) were fertile. These findings were consistent with those of Saad and Magzoub [77].

Our research revealed that hydatid cysts infected with the common hydatid seemed to have the characteristic form of a common hydatid cyst, including a germinal layer, a cuticular membrane, a fibrous tissue capsule, and cellular infiltration, all of which were identified in lung tissue afflicted by the infection. It was previously demonstrated by Dyab et al. [13] that the fibrous capsule was made up of a thick layer of connective tissue that was infiltrated with lymphocyte and plasma cell aggregates.

According to Khadidja et al. [64], the fibrous capsule is formed as a result of an inflammatory reaction between the host and the parasite.

The histopathological examination of the tissue sample in the current research demonstrated evidence of cellular response among the host tissue, represented by a fibrous layer infiltration with inflammatory cells. The lung tissues enclosing the hydatid cyst have indeed been noted to have extensive mononuclear infiltration [65]. However, Barnes et al. [66] observed a mixed inflammatory infiltrate in the outer membrane wall of lung hydatid cysts.

Substantial alveolar injury, including some alveoli emphysematous vascular congestions and hemorrhages, as well as atelectasis, were seen in the lungs of camels afflicted by hydatid cysts. Inflammatory cells typically invaded a hydatid cyst-infected liver and nodules developed, which included inflammatory cells, collagen fiber, and fibroblast. Severe bleeding, hepatocyte necrosis, and cytoplasmic expansion were also detected. Beigh et al. [62] and Anbu et al. [62,63] came to quite comparable findings.

Damage inflicted by hydatid cysts in the liver was comparable with that described in the literature [67]. According to [68], the examined liver cyst had caseated material that was entangled with dystrophic calcification and encircled by a fibrous capsule and mononuclear inflammatory cells. The proteolytic action of recruited inflammatory cells was hypothesized to degrade the extracellular matrix [69], leading to a loss of cord-like architecture in the hepatocytes of pathological liver sections. Additionally, the softer consistency of lung tissue may account for the higher frequency of hydatid cysts in lung tissue than those in liver. High reticuloendothelial cell numbers and a robust connective tissue reaction inside the organs suggest why just a few hydatid cysts were recovered from the livers and evolved to calcified cysts. A small-sized hydatid cyst had been located on the liver, and it had come to be postulated that the host’s immune response kept the cyst from expanding [70].

## 5. Conclusions

The findings of this investigation support the endemic nature of hydatidosis in camels. According to the findings of the study on hydatidosis in Egyptian camels. The prevalence of hydatidosis was higher in female older camels. The hydatid cysts were mostly observed in lung rather than liver, and the infection associated with high MAD level. It is advised that developing a surveillance program and hygienic measures can be helpful in lowering the incidence rate of the disease. Additionally, to protect infected organs (liver and lung) from being consumed by wild animals, infected animal carcasses should be destroyed.

## Figures and Tables

**Table 1:** Prevalence and factors associated with hydatid cyst infection in slaughtered camels.

Variable	Total Examined Camels	No. of Positive	%	*p* Value
Sex				
Male	82	15	18.29	0.269 *
Female	70	18	25.71	
Age				
<4 years	120	27	22.50	0.648 *
>4 years	32	6	18.75	
Total	152	33	21.7	

* The result was non-significant at *p* > 0.05.

**Table 2:** Parasitological characters and biochemical analysis of hydatid cyst in positive camels.

Animal	Organ	Cyst Status	Fluid Status	Fluid Color	Size (cm)	Fluid Amount (mL)	Sodium (mmol/L)	Potassium (mmol/L)	Calcium (mg/100 mL)	Phosphorus (mg/100 mL)
1	Lungs	Sterile	Watery	Clear	4	9	61.4	2	42	0.7
2	Lungs	Fertile	Thick	Turbid	6	10	60.3	0.3	33	1.3
3	Lungs	Fertile	Watery	Clear	4	8	35.9	1.8	9	4.5
4	Liver	Sterile	Watery	Clear	3	7	55	1.4	13	1
5	lungs	Sterile	Watery	Clear	8	15	44.4	1.8	66	1.4
6	Lungs	Fertile	Thick	Turbid	7	12	64.5	0.6	39	1.5
7	Lungs	Sterile	Watery	Clear	7	11	59	0.9	55	1.8
8	Liver	Sterile	Watery	Clear	2	5	50.1	1.4	13	0.9
9	Lungs	Fertile	Watery	Clear	6	12	40.5	1.5	71	2.5
10	Lungs	Fertile	Watery	Clear	7	16	34.8	1.7	62	1.3
11	Lungs	Fertile	Watery	Clear	8	16	48	1.8	60	1.2
12	Lungs	Fertile	Thick	Turbid	7	10	67.3	0.8	40	1.7
13	Liver	Sterile	Watery	Clear	3	6	52.7	1.3	11	1.1
14	Lungs	Fertile	Watery	Clear	8	15	40	1.6	60	1.3
15	Lungs	Fertile	Watery	Clear	5	10	72	2.2	50	0.8
16	Lungs	Fertile	Thick	Turbid	7	9	66.2	0.7	38	1.7
17	Lungs	Fertile	Watery	Clear	5	9	35.5	1.1	84	2
18	Lungs	Sterile	Watery	Clear	3.5	8	68.1	2.1	44	0.4
19	Lungs	Fertile	Watery	Clear	6	13	45.4	1.5	25	1.4
20	Lungs	Fertile	Watery	Clear	6	14	35.3	1.3	38	1.3
21	Liver	Sterile	Watery	Clear	2.5	5	43.7	1.7	19	1
22	Liver	Sterile	Watery	Clear	2	5	40.2	1.7	22	0.9
23	Lungs	Fertile	Watery	Clear	4	9	38.5	1.7	10	4.8
24	Lungs	Sterile	Watery	Clear	4.5	10	51	1.5	59	0.6
25	Liver	Sterile	Watery	Clear	2	4	69	0.7	39	0.4
26	Lungs	Fertile	Thick	Turbid	4	4	66.1	0.5	40	1.4
27	Lungs	Fertile	Thick	Turbid	8	6	62	0.4	33	1.3
28	Lungs	Sterile	Watery	Clear	5	11	48.3	1.7	40	0.9
29	Lungs	Fertile	Thick	Turbid	7	11	45.8	0.5	30	1.1
30	Lungs	Fertile	Watery	Clear	5	8	33.2	1.6	15	1.9
31	lungs	Sterile	Watery	Clear	5	12	40.1	1.5	57	1.5
32	Lungs	Fertile	Thick	Turbid	6	9	60.1	0.9	40	1.4
33	Lungs	Sterile	Watery	Clear	7	13	53.2	1.3	51	1.6

**Table 3:** Serum antioxidant activities (mean ± SE) in infected and control camels.

Animal	MDA (nmol/mL)	GSH (U/mL)	SOD (U/mL)	CAT (mM/L)
Infected	6.61 ± 19	3.32 ± 0.13	78.07 ± 0.96	113.52 ± 6.84
Control	1.96 ± 0.06	7.04 ± 0.12	122.64 ± 1.65	157.77 ± 1.48
*p* value	<0.0001 *	<0.0001 *	<0.0001 *	<0.0001 *

* The result was significant at *p* value < 0.05.

## Data Availability

This article contains all of the data that were created or analyzed throughout the investigation.
